# From Beer to Cheese: Characterization of Caseinolytic and Milk-Clotting Activities of Proteases Derived from Brewer’s Spent Grain (BSG)

**DOI:** 10.3390/foods13223658

**Published:** 2024-11-17

**Authors:** Maximiliano M. Villegas, Johana N. Silva, Florencia R. Tito, Claudia V. Tonón, Fernando F. Muñoz, Alfonso Pepe, María G. Guevara

**Affiliations:** 1 Biological Research Institute, National Scientific and Technical Research Council (CONICET), University of Mar del Plata (UNMDP), Mar del Plata 7600, Argentina; maximilianovillegas@mdp.edu.ar (M.M.V.); johanassilva@gmail.com (J.N.S.); ftito@mdp.edu.ar (F.R.T.); ctonon@mdp.edu.ar (C.V.T.); fermunoz81@gmail.com (F.F.M.); 2 Plant Physiology Group, Faculty of Agricultural Sciences, National University of Litoral, Esperanza 3080, Argentina; 3 H. Lee Moffitt Cancer Center and Research Institute, Tampa, FL 33612, USA

**Keywords:** circular economy, barley bioproducts, vegetable rennet, biomass sustainability, food innovation, enzymes

## Abstract

This study explores the extraction and characterization of proteolytic enzymes from brewer’s spent grain (BSG) and their potential as sustainable coagulants in the dairy industry. BSG samples from various beer types (Blonde Ale, IPA, Kölsch, Honey, and Porter) were obtained from two artisanal breweries in Mar del Plata, Argentina. Optimization of caseinolytic activity (CA) and protein extraction was conducted using a Plackett–Burman design, followed by a Box–Behnken design. Optimal protein concentration was achieved at intermediate pH and high temperature, while CA peaked at pH 8.0. The specific caseinolytic activity (SCA) varied among the extracts, with BSG3 showing the highest activity (99.6 U mg^−1^) and BSG1 the lowest (60.4 U mg^−1^). Protease inhibitor assays suggested the presence of aspartic, serine, metallo, and cysteine proteases. BSG3 and BSG4 showed the highest hydrolysis rates for α-casein (70% and 78%). For κ-casein, BSG1, BSG2, and BSG3 demonstrated moderate activity (56.5%, 49%, and 55.8), while BSG4 and BSG5 exhibited the lowest activity. Additionally, the milk-clotting activity (MCA) of BSG extracts was comparable to plant-based coagulants like *Cynara cardunculus* and *Ficus carica*. These findings highlight the potential of BSG-derived proteases as alternative coagulants for cheese production, offering a sustainable link between the brewing and dairy industries.

## 1. Introduction

Brewer’s spent grain (BSG) is the principal solid by-product of the brewing process, accounting for approximately 85% of the total waste produced in beer manufacturing [1]. This biomass, produced in vast quantities by breweries worldwide, has historically posed a waste management challenge [2]. However, recent research has highlighted the potential of BSG as a valuable raw material, given its nutrient-rich profile, which includes proteins, fibers, and phenolic compounds that make it suitable for numerous high-value applications [3]. The shift toward a circular economy, aiming to reuse and repurpose by-products, has brought BSG into the spotlight, underscoring its relevance in food, bioenergy, and biotechnology sectors [4]. This approach aligns with sustainable development goals by enabling industries to reduce waste and extend the lifecycle of natural resources, transforming BSG into a renewable input for various industries. Reusing waste like BSG multiple times exemplifies a compelling strategy to advance the circular economy, transforming industrial by-products into assets that reduce the environmental impact of the brewing industry [5].

During the brewing process, barley undergoes a series of enzymatic and thermal treatments during malting and mashing, stages that promote protein degradation. Enzymes, particularly proteases, break down storage proteins into smaller peptides and amino acids that facilitate yeast metabolism [6,7]. These BSG proteins, though partially denatured, retain significant functional properties even after the brewing process. Studies indicate that thermostable proteases in BSG maintain their proteolytic activity under various conditions, presenting an underexplored yet promising opportunity for biotechnological applications beyond brewing [8,9]. Such enzymes have attracted interest in food processing, especially for their robust activity under diverse pH and temperature conditions, making them suitable candidates for applications where resilience to processing conditions is critical [10].

One of the most promising applications for BSG-derived proteases lies in the dairy industry. The proteolytic activity of these enzymes can be harnessed as an alternative to traditional animal-derived coagulants used in cheese-making [11,12,13]. Traditional cheese coagulants, such as animal rennet, are derived from calves and are increasingly limited by ethical and supply challenges, prompting the search for plant-based or microbial alternatives [14,15]. Proteolytic enzymes, including aspartic and serine proteases, which demonstrate resilience to various thermal and chemical conditions, have the capacity to hydrolyze casein, a key protein in milk that undergoes structural changes to form cheese curds. Proteases that can selectively degrade casein subunits (especially κ-casein) are particularly valuable, as their action initiates curd formation by disrupting micelle structures in milk [16]

Despite substantial research into BSG’s nutritional and functional composition, the potential to harness BSG-derived proteases for high-value industrial applications, particularly in cheese production, remains underexplored [17,18]. This study addresses this gap by optimizing and characterizing the caseinolytic and milk-clotting activities (MCAs) of proteases extracted from BSG. The research focuses on assessing the enzymatic activity of BSG proteases on milk proteins, proposing these enzymes as plant-based coagulants capable of supporting sustainable dairy applications. Specifically, this study investigates their action on key milk caseins, aiming to identify optimal extraction conditions and understand the enzymes’ capabilities for use in dairy processing.

By advancing the understanding of BSG proteases, this research contributes to the growing body of knowledge on sustainable food innovation, aligning with the principles of a circular bioeconomy [19,20]. The outcomes could support the development of BSG-derived coagulants, offering a viable, natural alternative to animal rennet, thereby reducing reliance on animal-based coagulants and providing an eco-friendly solution for the dairy industry. Ultimately, this study represents a significant step in linking the brewing and dairy industries, demonstrating the feasibility of transforming a brewing by-product into a functional ingredient for artisanal cheese production. These findings not only reduce waste but also open pathways for developing functional food ingredients, enhancing the commercial value of BSG and promoting more sustainable, innovative food production practices.

## 2. Materials and Methods

### 2.1. BSGs Source

The BSGs were provided by the artisanal breweries “Antares” and “Cheverry” in the city of Mar del Plata, Argentina.

We designated the BSG used in this work as BSG1, derived from an Blonde Ale beer type, composed of Pilsner malt (87.5%) and Carapils malt (12.5%), sourced from Cheverry Brewery; BSG2, derived from an IPA beer type, which consisted of Pale malt (95.2%) and Crystal malt (4.8%); BSG3 from a Kölsch beer type, containing Pilsner malt (87.5%) and Carapils malt (12.5%); BSG4 from a Honey beer type, composed solely of Pale malt; and BSG5 from a Porter beer type, consisting of Pale Ale malt (75%), Crystal malt (11%), Chocolate malt (7%), black malt (2.5%), wheat malt (4.5%), and oat flakes (4.5%). BSG2, BSG3, BSG4, and BSG5 were provided by Antares Brewery.

### 2.2. Experimental Design

The Plackett–Burman design was used to determine the significant variables affecting enzyme extraction conditions from BSG. Six independent variables at two levels were evaluated: pH, temperature, homogenization time, dithiothreitol (DTT), Triton X-100, and CaCl_2_ concentrations. The variables and their levels are shown in Table 1. Two response variables were analyzed: protein concentration and caseinolytic activity. They were measured according to Section 2.5 and Section 2.8.

The significant variables were optimized using a Box–Behnken design (BBD). A BBD is an independent, rotatable quadratic design that does not include embedded factorial or fractional factorial points. The variable combinations are located at the midpoints of the edges of the experimental variable space and at the center point [21]. The variables studied and their levels are shown in Table 2. The design included three replicates at the center point.

### 2.3. Statistical Analysis

The results obtained from the Plackett–Burman and the Box–Behnken designs were analyzed and plotted using Minitab Software version 20.4 (Minitab LLC, 2021, State College, PA, USA) and the R software version 3.6.3 [22]. Analyses of variance were used to determine the factors that were significant at the 5% level for both designs, and Pareto charts were plotted. The responses obtained from the BBD experiments were analyzed using a response surface methodology (RSM) [23]. Second-order polynomial equations including interaction terms were used to build the models and obtain the coefficients. An analysis of variance was used to determine the significance of each coefficient.

### 2.4. BSG Protein Extracts

The different BSG protein extracts were obtained according to the experimental design used (Section 2.2). Different buffers (100 mM Acetate buffer pH 5, 100 mM Phosphate buffer pH 7 and 100 mM Tris-HCl buffer pH 9) were placed in a mixer cup in a 1:5 ratio (BSG:buffer) and supplemented with different concentrations of DTT (Sigma-Aldrich, Burlington, MA, USA), Triton X-100 (Fisher Scientific, Waltham, MA, USA), and CaCl_2_ (Sigma-Aldrich, Burlington, MA, USA). The mixture was homogenized through 2, 4, or 6 pulses of 30 s each, using an immersion blender (SL-SM6038WPN 600 W, Smartlife, Buenos Aires, Argentina). A water bath was used to fix the temperature to the corresponding value (30, 45, or 60 °C). Then, to obtain the protein extracts, the homogenates were centrifuged at 10,000× *g* for 20 min at 4 °C (Thermo Scientific Sorvall ST16R, Fisher Scientific, Waltham, MA, USA). Once the BSG extracts were prepared, they were sterilized by exposure to shortwave UV light in the UVC band (200–280 nanometers). The supernatant was then collected and stored at −20 °C until use.

### 2.5. Protein Determination

The sample protein content was determined using a bicinchoninic acid (BCA) method [24]. Bovine serum albumin (BSA) was used as the standard for calibration. The protein concentrations of the extracts were measured using an ELx800 ELISA plate reader (Biotek, Winooski, VT, USA), with absorbance at 540 nm. Since DTT could interfere with the BCA method [25], the extracts were previously dialyzed. Dialysis of the extract was carried out for 24 h in different buffers, which were replaced after 12 h. The dialyzed extracts were stored at −20 °C.

### 2.6. SDS-PAGE (Sodium Dodecyl Sulfate Polyacrylamide Gel Electrophoresis)

For protein profile analysis, SDS-PAGEs were performed using 12% (*w*/*v*) polyacrylamide gels under reducing conditions, according to the procedure of Laemmli [26]. Samples were dissolved (1:5) in a sample buffer containing 60 mM Tris-HCl buffer pH 6.8, 10% *v*/*v* glycerol, 2% SDS, 5% *v*/*v* 2-mercaptoethanol, and 0.05% *v*/*v* bromophenol blue, heated at 95 °C for 5 min and kept on ice to ensure denaturation. The loading volumes were 5 µL for molecular weight markers (MWM) and 20 µL for the samples. Electrophoresis was carried out at a constant voltage of 100 V during stacking and 120 V until the run was complete. After electrophoresis, the gels were stained with a staining solution containing Coomassie Brilliant Blue R-250 at room temperature for 12 h and subsequently destained with a destaining solution until protein bands were visible against a clear background. The molecular weight of the proteins was estimated by comparing the migration pattern of the bands to the MWM composed of a mixture of BSA (66 kDa), chicken ovalbumin (45 kDa), glyceraldehyde-3-phosphate dehydrogenase (36 kDa), carbonic anhydrase (29 kDa), bovine pancreas trypsinogen (24 kDa), and soybean trypsin inhibitor (20.1 kDa) (Sigma-Aldrich, Burlington, MA, USA). The gels were scanned and analyzed using the ImageJ software version 1.54h [27].

### 2.7. Milk-Clotting Activity of BSG1 Extract

The milk-clotting activity (MCA) of the BSG1 extract was evaluated as described by Lizardi-Jiménez, with slights modifications [28]. In total, 12 g of skim milk powder (San Regim, Sunchales, Argentina) was reconstituted in 100 mM of phosphate buffer with pH 6.5 containing 40 µL of 10 mM CaCl_2_ to a final volume of 2 mL. The capacity of the BSG extract to clot bovine milk was determined by observing the volume and texture of the clots formed after 24 h of incubation at 37 °C. A total of 1 mg mL^−1^ of commercial chymosin (Chy-Max, CHR-Hansen, Hoersholm, Denmark) served as the positive control, while 100 mM of phosphate buffer with a pH of 6.5 was used as the negative control. Two different milk/BSG1 ratios were analyzed: 1:1 and 1:2.

### 2.8. Caseinolytic Activity of BSGs Extracts

The caseinolytic activity (CA) of the BSG extracts was measured using total casein as a substrate, according to Anusha with some modifications [29]. In total, 450 µL of each BSG extract was incubated with 150 µL of 2% *w*/*v* casein (Sigma-Aldrich, Burlington, MA, USA) in 100 mM of the phosphate buffer with a pH of 6.5 at 37 °C for 5 h. The hydrolysis of casein was evaluated by SDS-PAGE, as described previously. One caseinolytic activity unit (U) was defined according to Equation (1):

(1) $$U=\frac{I_{casein+BSG}}{I_{casein}}\times 45$$

where I_(casein+BSG) is the intensity of the band corresponding to casein incubated with the BSG extract, and I_casein is the intensity of the band corresponding to casein alone, both calculated from the densitometry of the SDS-PAGE gels.

### 2.9. Influence of pH and Temperature on CA

The influence of pH on CA of BSG1 extract was determined according to de Farias [30] across a pH range of 3 to 9, using the following buffers: 100 mM acetic acid/acetate buffer (pH 3–5), 100 mM phosphate buffer (pH 6–7), and 100 mM carbonate/bicarbonate buffer (pH 8–9). The activity was determined as described in Section 2.8 and was expressed as a percentage relative to the sample exhibiting the highest enzymatic activity (relative activity).

The enzymatic behavior at different temperatures was assessed by measuring CA across a temperature range of 30 °C to 70 °C in 10 °C intervals [30]. The activity was expressed as a percentage relative to the sample exhibiting the highest enzymatic activity (relative activity).

### 2.10. Endopeptidases Inhibition Profile

Endopeptidases are classified into four main groups based on their active sites, according to the MEROPS database [31]: aspartic peptidases, serine peptidases, metallopeptidases, and cysteine peptidases. To determine the main peptidase types that contribute to the proteolytic activity of BSG extract, an inhibition profile was performed. The effect of specific inhibitors on the CA of the BSG1 extract was assessed by pre-incubating the samples with 40 mM pepstatin A (aspartic peptidase inhibitor), 1.5 mM phenylmethylsulfonyl fluoride (PMSF) (serine peptidase inhibitor), 5 mM ethylenediaminetetraacetic acid (EDTA) (metallopeptidase inhibitor), or 4 mM iodoacetamide (cysteine peptidase inhibitor) for 1 h at 37 °C. Then, the CA of the BSG1 extract was measured as described in Section 2.8.

Inhibitor solutions were prepared as follows: Pepstatin A (Sigma-Aldrich, Burlington, MA, USA) was prepared as a 1 mM stock solution by dissolving it in dimethyl sulfoxide (DMSO) and was used at a final concentration of 40 mM in the reaction mixture; PMSF (Sigma-Aldrich, Burlington, MA, USA) was prepared as a 100 mM stock solution in isopropanol, as it is unstable in aqueous solutions, and was added just before the assay to maintain activity; EDTA (Fisher Scientific, Waltham, MA, USA) was prepared as a 0.5 M stock solution by dissolving it in water and adjusting the pH to 8.0 with NaOH to ensure solubility; and finally, iodoacetamide (Sigma-Aldrich, Burlington, MA, USA) was dissolved in water to create a 100 mM stock solution and was added immediately before use due to its light sensitivity. All stock solutions were stored at −20 °C, except PMSF, which was ideally prepared fresh, to prevent degradation.

### 2.11. Hydrolysis of the Bovine Casein Subunits

BSG’s endopeptidases activity on bovine α-, β-, and k-casein subunits was determined according to the method described by Pontual [32]. Commercial bovine α_s_-, β-, and κ-casein subunits (Sigma-Aldrich, Burlington, MA, USA) with molecular weights of 22–25, 24, and 19 kDa, respectively, were dissolved in 100 mM of the phosphate buffer with a pH of 6.5 to a final concentration of 0.8 mg mL^−1^ for α_s_- and β-casein and 0.65 mg mL^−1^ for κ-casein. Each subunit was independently incubated with the different BSG extracts in 100 mM of the phosphate buffer with a pH of 6.5 at 37 °C for 6 h. The reaction was stopped by adding a sample buffer. Digestion products were analyzed with SDS-PAGE as described previously. Hydrolysis percentage was calculated from the SDS-PAGE densitometric analysis, considering 100% hydrolysis as the complete disappearance of the casein bands. A total of 1 mg mL^−1^ of commercial chymosin (Chy-Max, CHR-Hansen, Hoersholm, Denmark) was used as control.

## 3. Results and Discussion

The brewing process significantly alters the protein content of barley grains [8,33]. Mashing, the first key step in brewing, continues the enzymatic degradation initiated during malting, with around 90% of endoproteases surviving the kilning process and remaining active during mashing, facilitating further protein breakdown into peptides and amino acids [8,33]. The hydrolytic enzymes involved in this degradation, mainly endoproteases and carboxypeptidases, are produced during malting and act to cleave proteins into smaller peptides and free amino acids. Jones et al. and Osman et al. demonstrated that endoproteases remain highly active, into the wort, at approximately 38 °C during the protein rest phase of mashing, but their activity declines rapidly when the temperature increases to 70 °C, where starch hydrolysis occurs and proteases are inactivated [8,34]. On the other hand, Rizvi et al. isolated and characterized proteases thermostables, from Canadian two-row spring malting barley lines, after kilning process (air drying at 40–60 °C, followed by a gradual temperature increase to 85–95 °C) [9].

Despite substantial research on protease activity during malting and mashing, there is a critical gap in the literature regarding the residual activity of endoproteases in BSGs after mashing, representing a promising area for further investigation.

### 3.1. Finding Significant Variables That Affect Extraction of Endopeptidases with Caseinolytic Activity from BSG

To identify the significant variables influencing the extraction of active endopeptidases from BSG, the Plackett–Burman experimental design [35] was used. This approach allowed us to systematically evaluate multiple factors simultaneously, providing insight into which variables have the most substantial impact on enzyme activity. The results obtained from this design are critical for optimizing the extraction process, ensuring maximum yield and efficiency in subsequent studies [36,37,38]. While the Plackett–Burman design does not differentiate between main effects and interaction effects, it effectively identifies factors with significant impacts by comparing the response differences between the two levels of each factor [39].

Table 3 outlines the screening design used and the associated response values. The response variables assessed were caseinolytic activity and protein concentration in the extracts.

**Figure 1:**
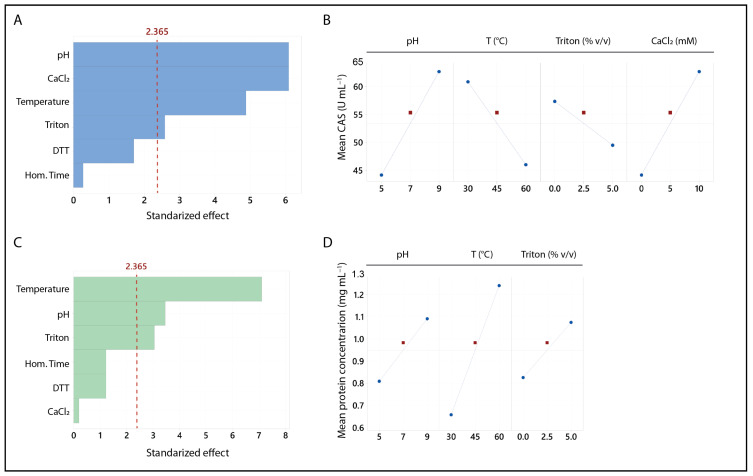
Pareto (**A**,**C**) and main effect plots (**B**,**D**) from the Plackett–Burman design analysis. The six independent variables examined were pH, temperature, homogenization time, and the concentrations of Triton X-100, DTT, and CaCl_2_. The blue plots represent CA, while the green plots correspond to protein concentration. Variables with values exceeding the threshold indicated by the dashed red line are considered significant.

The analysis of the Plackett–Burman design revealed that pH, temperature, Triton X-100, and CaCl_2_ concentration had a significant impact on CA (Figure 1A). Higher CA was associated with elevated pH and CaCl_2_ concentration and lower temperature and Triton X-100 concentration (Figure 1B). For the protein concentration, the significant variables were temperature, pH, and Triton X-100 concentration, all of which were positively correlated with the response (Figure 1C,D).

To further optimize the significant variables that maximize both CA and protein concentration, the BBD was analyzed using the RSM [40]. Table 4 presents the selected variables, their levels, and the corresponding responses obtained. The upper and lower limits for each variable were determined based on the results of the Plackett–Burman design analysis. A full quadratic model, including interaction terms, was employed to analyze the data. The two responses evaluated were CA and protein concentration.

**Figure 2:**
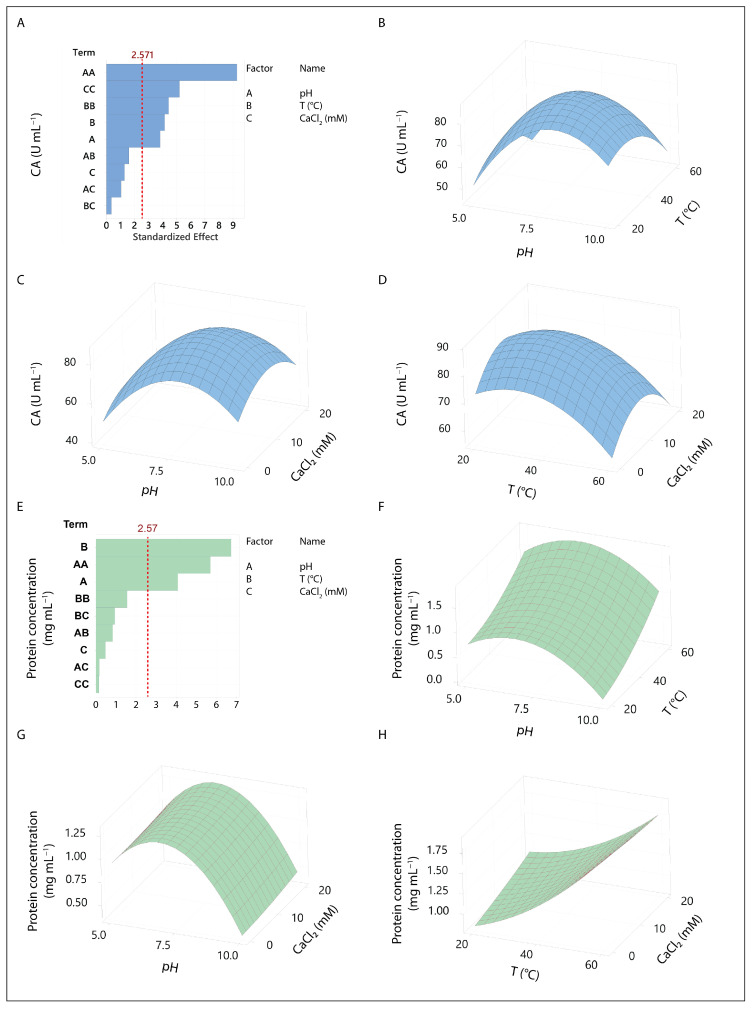
RSM analysis of the BBD. Two response variables were evaluated: CA and protein concentration. The independent variables identified as significant in the screening design (pH, temperature, and CaCl_2_ concentration) were selected for the BBD. Subpanels (**A**,**E**) display the significant terms for each model, with values exceeding the threshold indicated by the dashed red line considered significant. (**B**–**D**) depict the 3D CA response surfaces, showing the interactions between two independent variables while holding the third at its optimal level. (**F**–**H**) depict the 3D protein concentration response surfaces, showing the interactions between two independent variables while holding the third at its optimal level.

Figure 2 presents the RSM analysis of the BBD. The variables that positively influenced CA and protein concentration (pH, temperature, and CaCl_2_ concentration) were selected for this design.

Figure 2A highlights the significant terms in the CA analysis. All three quadratic terms were significant, and the linear terms for pH and temperature were also significant (*p* < 0.05). The resulting model is represented in Equation (2). The lack of fit is non-significant (*p* = 0.67), and the adjusted R^2^ value of 0.91 indicates that the model explains 91% of the variance.

(2) $$\begin{array}{c} CA=-171.4+64.65pH+2.169T+1.325CaCl_{2}-3.360pH^{2}-0.025T^{2}-\\ 0.1175CaCl_{2}^{2}-0.07pH\cdot T+0.09pH\cdot CaCl_{2}+0.0038T\cdot CaCl_{2} \end{array}$$

Figure 2E presents the significant terms in the analysis for protein concentration (PC). The linear terms for pH and temperature were significant (*p* < 0.05), and the quadratic term for pH was also significant. The resulting model is represented in Equation (3). The lack of fit was non-significant (*p* = 0.37), and the adjusted R^2^ of the model was 0.87, indicating that the model explains 87% of the variance. In both models (Figure 2A,E), none of the interaction terms were significant.

(3) $$\begin{array}{c} PC=-2.86+1.138pH-0.0152T+0.0126CaCl_{2}-0.0875pH^{2}+0.000374T^{2}+\\ 0.000155CaCl_{2}^{2}+0.00155pH\cdot T+0.00064pH\cdot CaCl_{2}-0.000434T\cdot CaCl_{2} \end{array}$$

The optimal values found for the variables evaluated for both responses are shown in Table 5. Optimal protein concentration conditions were different from those corresponding to CA. The response surface plots for protein concentration (Figure 2F–H) reveal that pH was the only significant quadratic term, while all quadratic terms were significant for CAs (Figure 2B–D). The differences in the response surfaces indicate that the maximum protein concentration was obtained at an intermediate pH and maximum temperature, with no significant effect from CaCl_2_ concentration. In contrast, for CA, the highest values were observed at intermediate levels of all independent variables.

The maximum protein extraction occurred at pH 7.0 and 60 °C. Similar conditions have been reported for other BSG extracts obtained involving physical methods such as sonication [39,41], suggesting that neutral pH and elevated temperature optimize protein extraction from BSG [10]. However, the model for CA shows that to achieve significant CA in BSG extracts, the pH should be around 8. Figure 2B,C illustrate that caseinolytic activity decreases as the pH deviates from this value. Although the temperature and CaCl_2_ concentration were also significant, their effects were smaller and less critical compared to pH (Equation (2)). Based on these results (Table 5), the conditions of pH 8, room temperature, and 10 mM CaCl_2_ were selected for obtaining the BSG extracts used in this study.

The use of the BBD for optimizing the extraction process was effective in maximizing enzyme activity from the BSG. Similar methodologies have been employed in other studies to optimize enzyme extraction from plant sources [36,42,43]. This statistical approach not only improved enzyme yield but also ensured that BSG extracts could be utilized in industrial-scale applications.

### 3.2. Characterization of CA of BSG1 Extract

**Figure 3:**
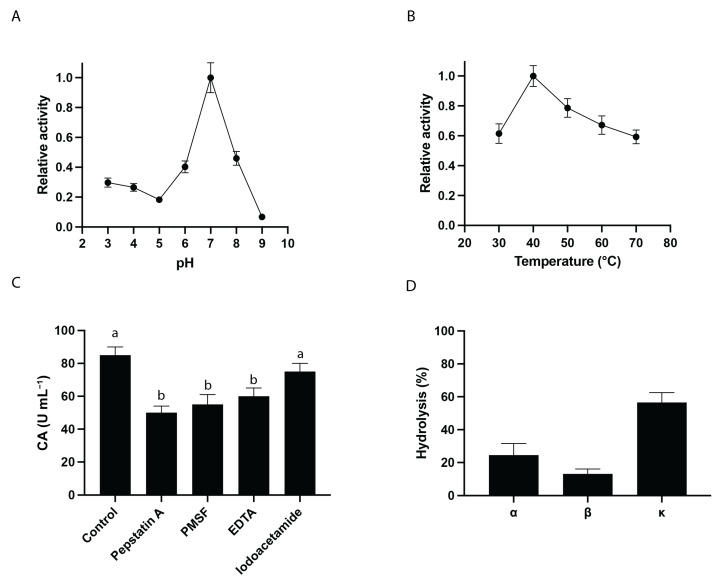
Biochemical characterization of CA of BSG1. (**A**) pH dependence: Relative activity of the BSG extract was measured across different pH values using citrate buffer (pH 3, 4, 5), phosphate buffer (pH 6, 7, 8), and Tris-HCl buffer (pH 9). (**B**) Temperature dependence: The relative activity of the extract was assessed at various temperatures using 100 mM phosphate buffer at pH 6.5. (**C**) Endopeptidases inhibition profile: Inhibitory effects on CA of BSG protein extracts of 40 mM Pepstatin A (aspartic peptidase inhibitor), 1.5 mM PMSF (serine peptidase inhibitor), 5 mM EDTA (metallopeptidase inhibitor), and 4 mM Iodoacetamide (cysteine peptidase inhibitor) were analyzed. CA values for each condition were calculated relative to BSG without inhibitors (control), as described in Section 2 Materials and Methods. (**D**) Specific caseinolytic activity (SCA): The SCA of BSG endopeptidases on bovine α-, β- and κ-casein subunits was determined as indicated in the Section 2 Materials and Method. Hydrolysis percentages were calculated using the SDS-PAGE densitometric analysis, considering 100% hydrolysis as the complete disappearance of the casein bands. Bars represent the standard deviation of three independent assays. Different letters indicate statistically significant differences (*p* < 0.05).

The CA of the BSG extract from Blonde Ale-type beer (Cheverry Brewery), named BSG1 and obtained under the optimum conditions described above, was assessed. Results obtained are shown in Figure 3.

The effect of pH on relative activity of BSG1 extract shows a clear trend, with the enzyme displaying its highest activity at a neutral pH of 7 (Figure 3A). As the pH moves away from neutrality, both in more acidic and basic conditions, the enzyme’s activity declines. In acidic conditions (pH 3 to 5), the relative activity decreases progressively, with the lowest activity observed at pH 5. In basic conditions (pH 8 to 9), there is a similar drop in activity, with the lowest activity at pH 9. During mashing, protease softens the outer layer of the kernel by breaking down the cell wall proteins, which exposes the starch to mashing enzymes, thereby improving both the mashing process and wort fermentability [44]. While proteases from malted barley typically function best in acidic conditions, it has been found to remain active even at higher pH levels, potentially up to pH 10 [44,45].

The temperature-dependent activity of the BSG1 extract (Figure 3B), as indicated by its proteolytic activity profile, aligns with findings from studies on proteases derived from plant-based or industrial byproducts [15,16,46]. The optimal BSG1 CA around 40 °C, followed by a sharp decline beyond this temperature, is a typical pattern observed in enzymes sensitive to thermal denaturation. Similar observations were made in studies of plant-derived proteases, where moderate proteolytic activity was recorded at lower temperatures, peaking between 35 °C and 45 °C, followed by a decrease due to protein unfolding or denaturation [47]. This thermal behavior is crucial for applications in brewing and food processing, where temperature control ensures maximal enzyme efficiency without compromising stability.

To identify the types of proteolytic enzymes present in the BSG1 extract, the effect of protease inhibitors on the CA was analyzed. Results showed that the caseinolytic activity of the BSG extract was partially inhibited by pepstatin A (50%), PMSF (56%), EDTA (61%), and Iodoacetamide (78%) (Figure 3C). BSG1 extract shows that multiple classes of proteases contribute to its enzymatic profile, suggesting the presence of aspartic, serine, metallo, and cysteine proteases in varying degrees. Additionally, the type of proteolytic enzymes determined in the BSG1 extract reveals both similarities and key differences, with proteins previously described in barley malted grains and wort [48,49,50,51,52], particularly regarding the roles of aspartic, serine, metalloproteases, and cysteine proteases. These proteases are crucial in the breakdown of storage proteins during mashing, with implications for the solubilization of protein and the production of free amino nitrogen, both of which influence wort composition and beer quality. In comparison, Jones and Budde examined the roles of proteases in barley malt during mashing and found that metalloproteases had the most significant effect on protein solubilization, particularly at pH 6.0 [53]. Their experiments showed that metalloproteases, inhibited by o-phenanthroline, played a larger role in solubilizing proteins than cysteine proteases, which were expected to be more active at acidic pH. This observation is consistent with the BSG data, where metalloproteases also played a prominent role, as indicated by EDTA inhibition. However, Jones and Budde noted that EDTA often increased protease activity in their barley malt system, which contrasts with the inhibitory effect observed in BSG extract [53]. This discrepancy could be explained due to the different extraction conditions or substrates used, with EDTA potentially having variable impacts depending on the proteolytic environment and the metal ions involved. On the other hand, the partial inhibition by pepstatin A of BSG1 extract confirmed the presence of aspartic proteases, consistent with their secondary but important role observed in barley mashes at acidic pH levels (pH 3.8). This supports earlier research by Brijs et al., which highlighted the key role of aspartic proteases in hydrolyzing storage proteins during seed germination [54]. These proteases may also contribute to protein degradation in barley during both malting and mashing. Serine proteases also exhibit similar behavior to aspartic and metalloproteases in the BSG1 extract. Similar results were previously reported for Jones and Budde et al., where serine proteases were active at higher pH (pH 8.0). The role of serine proteases in BSG is comparable to that in barley malt mashes, where they contribute to protein solubilization during mashing. These enzymes likely play a crucial role in processing proteins at more alkaline pH levels, helping to degrade proteins that resist the action of other proteases under standard mashing conditions. A key distinction between the studies is the relatively minor role of cysteine proteases in the BSG1 extract, as evidenced by the weaker inhibition with iodoacetamide. This is consistent with previous findings, where cysteine proteases had a limited effect on protein solubilization, even at acidic pH levels [53]. The results suggest that in both BSG and malt mashes, cysteine proteases may not be as significant as previously assumed, particularly when compared to the more dominant roles of metalloproteases and aspartic proteases.

The analysis of the CA on bovine casein subunits of BSG1 (Figure 3D) showed selective proteolysis of α-, β-, and κ-casein subunits under conditions resembling industrial coagulation (pH 6.5, 37 °C). κ-casein underwent the most extensive hydrolysis (56%), followed by α-casein (25%) and β-casein (13%). The varying degrees of hydrolysis observed in α-casein, β-casein, and κ-casein are in line with the distinct roles these subunits play in casein micelle structure. β-casein is known for its high hydrophobicity, which makes it more resistant to enzymatic hydrolysis, explaining the lower hydrolysis percentage observed in the BSG1 extract [55]. In contrast, α- and κ-casein subunits are more accessible to proteolytic enzymes due to their structural positions and solubility characteristics, resulting in higher degradation rates. This pattern has been observed in other studies investigating plant-based coagulants, where proteases from cardoon and papaya showed preferential hydrolysis of κ-casein and α-casein, facilitating micelle destabilization and curd formation [16]. Our analysis of the protease types within BSG extracts revealed the presence of serine proteases, which play a crucial role in milk clotting and casein hydrolysis. Notably, these serine proteases exhibit properties similar to those of chymotrypsin and elastase-2, both known for their precise substrate specificity and controlled hydrolysis capabilities. This similarity suggests that BSG-derived serine proteases can effectively target specific casein subunits, particularly κ-casein, in a manner that facilitates milk coagulation without excessive proteolysis. The ability to achieve controlled hydrolysis enhances the functional value of BSG proteases as coagulants. This property not only preserves the texture and flavor of the final product but also aligns with commercial requirements for cheese production by allowing precise regulation of curd formation [56].

**Figure 4:**
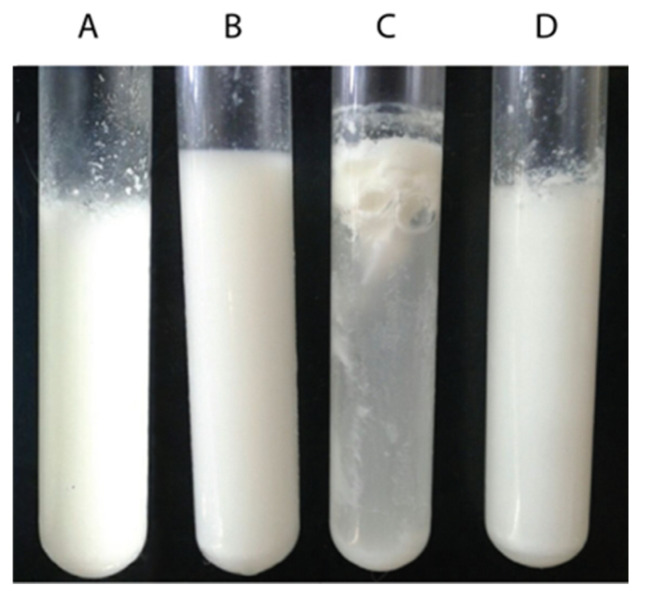
Milk-clotting activity of BSG proteases. Commercial skim milk was dissolved in a phosphate buffer (pH 6.5) containing 40 µL of 10 mM CaCl_2_, in a final volume of 2 mL, and incubated at 37 °C for 24 h. The following conditions were tested: (**A**) chymosin served as a positive control, (**B**) phosphate buffer with a pH of 6.5 was used as a negative control, (**C**) commercial skim milk in phosphate buffer with a pH of 6.5 with 10 mM CaCl_2_ and BSG extract at a 1:2 ratio, and (**D**) 1:1 ratio.

In order to evaluate the capacity of BSG1 extract to coagulate bovine milk for cheese-making, an MCA test was performed as detailed in the Section 2 Materials and Methods. This assay allows for the evaluation of the first stage of cheese-making, called enzymatic coagulation, which directly affects the quality of the final product [57]. Results shown in Figure 4 demonstrate that BSG1 extract exhibits MCA in a dose-dependent manner. After 24 h of incubation, no significant differences were observed in the volume or texture of the clots formed using chymosin or BSG1 extract at a 1:1 (milk/BSG) ratio.

The biochemical characterization of the CA and MCA of the BSG1 extract highlights its potential as an alternative coagulant in artisanal cheese production, and provides a sustainable option that utilizes the functional properties of brewing-industry byproducts. These findings align with the growing interest in plant-based and waste-derived enzymes, which offer both environmental benefits and novel functionalities for industrial applications [58,59,60]. However, further studies will be necessary to investigate the organoleptic characteristics of the resulting curds in greater detail, in order to fully assess this potential.

### 3.3. Comparative Analysis of BSGs Derived from Different Beer Styles: Impact on CA and MCA

To investigate whether the CA and MCA of BSG extracts are influenced by the type of malt used, both activities were measured using BSG derived from various beer styles, each with different malt composition and malting processes. The different types of BSG used and their composition were previously described in the Section 2 Materials and Methods and were abbreviated as BSG1, BSG2, BSG3, BSG4, and BSG5.

**Figure 5:**
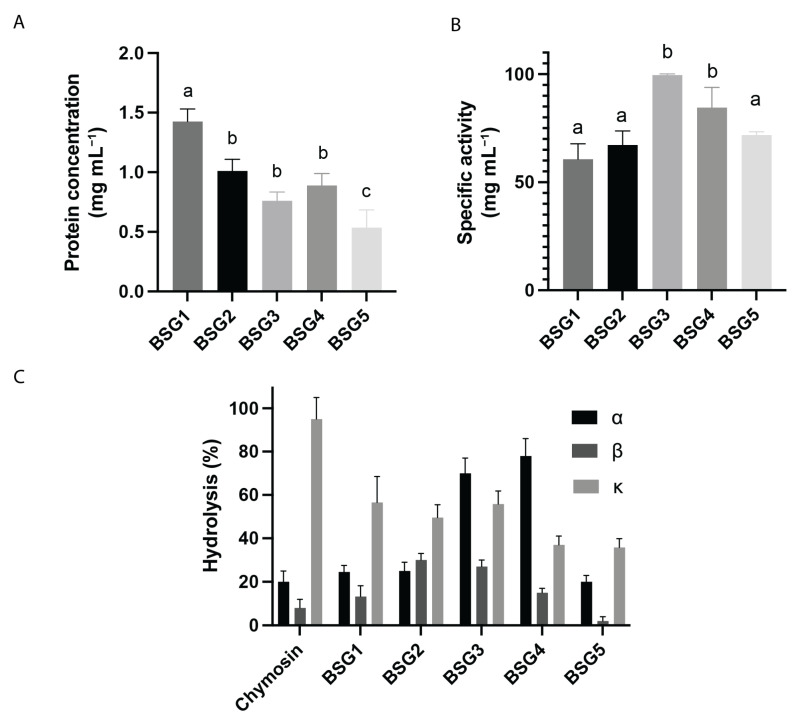
Comparative analysis of BSG extracts: protein concentration, specific caseinolytic activity, and activity on different casein subunits. (**A**) Protein concentration (mg mL^−1^) of different BSG extracts evaluated. (**B**) Specific caseinolytic activity of BSG extracts from different styles of beer. (**C**) Proteolytic activity on different casein subunits of BSG extracts from different styles of beer. Bars represent the standard deviation of three independent assays. Different letters indicate statistically significant differences (*p* < 0.05).

To determine the specific caseinolytic activity (SCA) of the different BSGs, the protein concentration and the CA of each BSG extract was measured using casein as the substrate. The protein concentration of the BSG extracts was determined, and results are shown in Figure 5A. The BSG1 extract showed the highest protein concentration (1.4 mg mL^−1^), while BSG2, BSG3, and BSG4 extracts presented moderate protein concentration values with 1.01, 0.89, and 0.76 mg mL^−1^, respectively. The value of BSG1 extract was significantly higher than the one obtained for BSG5 extract, which presented a protein concentration of 0.64 mg mL^−1^ extract.

Figure 5B shows the SCA of the BSG extracts tested. BSG2 exhibited moderate activity with an average SCA of 67.2 U mg^−1^, while BSG3 showed the highest activity with a value of 99.6 U mg^−1^, indicating that this extract contains the most active proteases. BSG4 also displayed a significant specific activity of 84.5 U mg^−1^. In contrast, BSG5 had a moderate specific activity of 71.8 U mg^−1^. Although the BSG1 extract displayed the highest protein content, its SCA value was the lowest (60.4 U mg^−1^), indicating that only a part of these proteins corresponds to active proteases. These results highlight significant differences in the enzymatic profiles of the extracts, suggesting their dependence with the BSG malt composition and malting processes.

The specificity of BSG extracts on bovine casein subunits (α-, β-, and κ-casein) was analyzed for the BSG extracts and for chymosin (Figure 5C). For α-casein, the BSG3 and BSG4 extracts exhibited the highest hydrolysis rates, at 70% and 78%, respectively. These values are notably higher than those for BSG1 (25%), BSG2 (25%), and BSG5 (20%), which showed activities similar to chymosin (20%). In the case of β-casein, the hydrolysis percentages were generally lower across all samples compared to α-casein. The BSG3 extract demonstrated the highest activity (30%), followed by BSG2 (27%), BSG4 (15%), and BSG1 (13%). Chymosin and BSG5 had the lowest activities, at 8% and 2%, respectively, indicating that β-casein is less susceptible to hydrolysis by these extracts. For κ-casein, the chymosin extract exhibited the highest mean hydrolysis rate at 95%, indicating strong proteolytic activity against this subunit. The BSG extracts displayed varying activities, with BSG1 (56.5%), BSG2 (49%), and BSG3 (55.8%) showing moderate hydrolysis, while BSG4 (37%) and BSG5 (35.8%) had the lowest activities.

In summary, the data indicate that BSG extracts exhibit different levels of proteolytic activity depending on the casein subunit. The BSG3 and BSG4 extracts were most effective against α-casein, while β-casein was less susceptible to hydrolysis by both chymosin and the BSG extracts, with the BSG3 extract being the most effective among the BSG samples. These variations may result from the different brewing processes used by artisanal beer producers, leading to different enzymatic profiles in the BSG extracts. Such differences should be considered when using BSG for artisanal cheese production.

**Figure 6:**
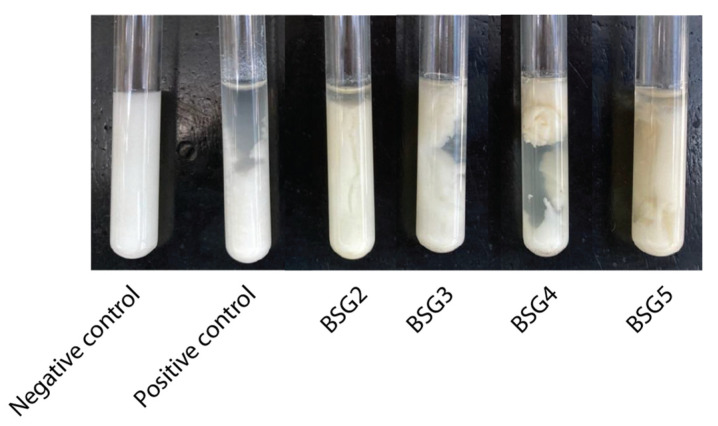
MCA of BSG extracts from different types of beer: 1 mL of extract was added to 1 mL of 12 % *w*/*v* commercial skim milk dissolved in 100 mM of the phosphate buffer with a pH of 6.5 containing 40 μL of 10 mM CaCl_2_, in a final volume of 2 mL, and incubated at 37 °C for 24 h. Chymosin was used as the positive control, and 100 mM of the phosphate buffer with a pH of 6.5 was used as the negative control.

To evaluate the MCA of the different BSG extracts, clots were formed for each extract as outlined in the Section 2 Materials and Methods section (Figure 6).

All tested BSG extracts successfully induced clot formation (Figure 6), demonstrating the milk-clotting capability of the proteases present. However, noticeable differences in clot texture were observed among some of the BSG extracts compared to that obtained with chymosin. The nature of the coagulant plays a significant role in defining clot characteristics, as it can influence micellar aggregation and affect both the rate of gel hardening and its final firmness, which in turn impacts the curd’s drainage properties, moisture content, texture, and cheese flavor [61,62]. The difference in the general characteristics of the clots produced compared to chymosin could be primarily due to the specific activities of each peptidase on the different casein subunits [63].

The MCA of the BSG extracts described was comparable to that of plant-derived enzymes, such as those from *Cynara cardunculus* and *Ficus carica*, as demonstrated in studies by Shah et al. [11] and Nitu et al. [64]. In recent years, plant-based coagulants have garnered interest as alternatives to calf rennet, particularly in regions like the Mediterranean. The ability of BSG extracts to clot milk without inducing excessive proteolysis presents a notable advantage over other plant coagulants, such as those from Ficus and Cynara, which are often associated with bitter flavors and suboptimal cheese texture.

## 4. Conclusions

This study successfully highlights the potential of BSG as a source of proteolytic enzymes with significant caseinolytic and milk-clotting activities, offering a sustainable alternative to traditional dairy coagulants. By optimizing the extraction and characterization of BSG-derived proteases, this research addresses an important gap in the valorization of brewery by-products, demonstrating how BSG can be repurposed for high-value applications within the dairy industry. The findings underscore the feasibility of integrating BSG proteases as plant-based coagulants, which could replace or supplement animal rennet in artisanal cheese production. This innovation not only reduces industrial waste but also advances circular-economy practices, linking the brewing and dairy sectors in a sustainable and commercially valuable manner. This study’s insights contribute to sustainable food processing and pave the way for future research on the scalability and sensory impacts of BSG-derived coagulants in cheese-making.

## Figures and Tables

**Table 1:** Plackett–Burman-evaluated variables and their levels.

Variable	Levels	
	−1	+1
pH	5	9
Temperature (°C)	30	60
Homogenization time (min)	1	3
DTT (mM)	0	10
Triton X-100 (%*v*/*v*)	0	5
CaCl_2_ (mM)	0	10

**Table 2:** BBD-evaluated variables and their levels.

Variable	Levels	
	−1	+1
pH	5	10
Temperature (°C)	20	60
CaCl_2_ (mM)	0	20

**Table 3:** Plackett–Burman design for six factors and their responses.

Run	pH	T (°C)	Homogenization Time (min)	Triton (%*v*/*v*)	DTT (mM)	CaCl_2_ (mM)	CA (U mL^−1^)	Protein Concentration (mg mL^−1^)
1	9	30	3	0.0	0	0	61	0.80
2	9	60	1	5.0	0	0	42	1.60
3	5	60	3	0.0	10	0	31	1.00
4	9	30	3	5.0	0	10	83	0.90
5	9	60	1	5.0	10	0	43	1.30
6	9	60	3	0.0	10	10	65	1.20
7	5	60	3	5.0	0	10	45	1.50
8	5	30	3	5.0	10	0	33	0.60
9	5	30	1	5.0	10	10	51	0.55
10	9	30	1	0.0	10	10	82	0.75
11	5	60	1	0.0	0	10	50	0.85
12	5	30	1	0.0	0	0	55	0.35
13	7	45	2	2.5	5	5	55	0.95
14	7	45	2	2.5	5	5	51	1.10
15	7	45	2	2.5	5	5	60	0.90

**Table 4:** BBD and their responses. The significant variables that positively affected CA and protein concentrations were selected.

Run	pH	T (°C)	CaCl_2_ (mM)	CA (U mL^−1^)	Protein Concentration (mg mL^−1^)
1	5	20	10	50	0.82
2	10	20	10	70	0.12
3	5	60	10	45	1.38
4	10	60	10	51	0.99
5	5	40	0	49	1.03
6	10	40	0	55	0.48
7	5	40	20	45	0.87
8	10	40	20	60	0.39
9	7.5	20	0	75	0.66
10	7.5	60	0	60	1.87
11	7.5	20	20	65	1.08
12	7.5	60	20	53	1.95
13	7.5	40	10	85	1.08
14	7.5	40	10	90	1.38
15	7.5	40	10	80	1.22

**Table 5:** Optimal conditions for CA and protein concentration determined by RSM.

	CA	Protein Concentration
pH	7.9	7.0
T (°C)	33	60
CaCl_2_ (mM)	9	Non significant

## Data Availability

The original contributions presented in the study are included in the article, further inquiries can be directed to the corresponding authors.

## Abbreviations

BSG	brewer’s spent grain
BBD	Box–Behnken design
RSM	response surface methodology
CA	caseinolytic activity
MCA	milk-clotting activity
BCA	bicinchoninic acid
BSA	bovine serum albumin
MWM	molecular weight markers
PMSF	phenylmethylsulfonyl fluoride
EDTA	ethylenediaminetetraacetic acid
DMSO	dimethyl sulfoxide
SCA	specific caseinolytic activity
